# Benefits of Advance Oxytocin Preparation Could Extend to the Newborn

**DOI:** 10.9745/GHSP-D-15-00156

**Published:** 2015-05-20

## Abstract

Advance preparation of oxytocin not only facilitates rapid administration after delivery to prevent postpartum hemorrhage but also could free health workers to provide immediate neonatal resuscitation to non-breathing newborns within the critical 1-minute time window.

See related article by Moucheraud.

Details matter. To reliably ensure good care, health workers need clearly defined processes, including provisions to ensure reliable and timely availability of all needed supplies and equipment. The paper by Moucheraud and colleagues in this issue of GHSP takes a close look at a particular service delivery setting, documenting current practices with regard to routine administration of oxytocin immediately after birth for preventing hemorrhage.^1^ Specifically, they looked at the extent to which timely dosing was facilitated by having the oxytocin injection drawn up in advance.

According to guidelines released in 2006 by the International Confederation of Midwives (ICM) and the International Federation of Gynaecology and Obstetrics (FIGO), oxytocin is to be given within 1 minute of birth. The study by Moucheraud et al. assessed performance against this standard. The investigators found that having the injection drawn up in advance increased the proportion of cases in which oxytocin was delivered within this interval and shortened mean time to injection.

Although this timing threshold is arbitrary for maternal outcomes (and has not been retained in the current World Health Organization guidelines^2^), streamlining the process and ensuring timely injection, with high reliability, is important for other reasons. In many country settings with high maternal and newborn mortality, many births are attended by only a single skilled health worker who is responsible for care of both the mother and the newborn. The most common life-threatening complication around the time of labor and delivery is birth asphyxia. In approximately 1 in 20 births, the baby requires help to initiate breathing, and this needs to happen within a critical time window.^3^ Under the Helping Babies Breathe newborn resuscitation initiative, health workers are trained on the importance of helping the baby to breathe within the “Golden Minute” after birth. The greater the delay, the higher the probability of death.

Having oxytocin at hand, already drawn up in a syringe when the woman is still in active labor, means that administering the drug takes mere seconds, so that this needn’t get in the way of giving non-breathing newborns the immediate attention required. Advance preparation of the oxytocin should be an explicit operational standard, as one of various preparations that, if done reliably (helped by use of checklists), can ensure smooth, streamlined provision of key elements of care for both mother and newborn, even in situations where complications arise. *–Global Health: Science and Practice*
